# Potentially inappropriate medication use in older adult patients living with cancer using the Beers, STOPP, GO-PIM and Chinese criteria: A retrospective cross-sectional study

**DOI:** 10.1097/MD.0000000000047871

**Published:** 2026-03-13

**Authors:** Yulong Liu, Qiao Su, Dong Wu, Xiaodong Li, Xiaowu Wang

**Affiliations:** a Department of Pharmacy, Yingshang People’s Hospital, Fuyang, PR China; b Department of Medical Oncology, Yingshang People’s Hospital, Fuyang, PR China; c Department of Pharmacy, Fuyang People’s Hospital, Fuyang, PR China; d Department of Pharmacy, Huaibei People’s Hospital, Huaibei, PR China; e Department of Clinical Laboratory, The Second People’s Hospital of Fuyang City, Fuyang Infectious Disease Clinical College of Anhui Medical University, Fuyang, PR China.

**Keywords:** cancer, older adults, polypharmacy, potentially inappropriate medication

## Abstract

Due to the complexity of medication use in older adult patients living with cancer, the risk of potentially inappropriate medications (PIMs) was significantly increased, yet evaluation criteria vary. Therefore, this study aimed to detect the PIMs use by the 2023 American Geriatrics Society (AGS) Beers criteria, the 2023 Screening Tool of Older Persons’ Prescriptions (STOPP) criteria, the geriatric oncology potentially inappropriate medication (GO-PIM) scale, and the 2024 Chinese criteria in older adult patients living with cancer and compare the prevalence of PIMs and the accordance between the 4 PIM criteria; and further to explore related risk factors for PIMs according to the 4 criteria. This retrospective study included 484 older adult patients living with cancer. PIMs were analyzed based on the 4 criteria. The consistency of the evaluation results was compared between the 4 criteria, and influencing factors for PIMs were analyzed using multivariate logistic regression. The incidence of PIMs in older adult patients living with cancer was high in our study, with certain differences in consistency among the 4 criteria. The 2024 Chinese criteria were the most sensitive for identifying PIMs, and polypharmacy was the main influencing factor for the occurrence of PIMs in all criteria. It is necessary to strengthen medication therapy management for older adult patients living with cancer. The prevalence of at least one PIM identified by the 4 criteria ranged from 42.15% to 71.07%. The drugs with the highest incidence were metoclopramide, medroxyprogesterone, and cimetidine. The kappa statistics for the 2023 AGS/Beers criteria with the 2024 Chinese criteria, the GO-PIM scale and 2024 Chinese criteria indicated a good concordance (κ = 0.788 and 0810). The consistency between the 2023 AGS/Beers criteria and the 2023 STOPP criteria, the GO-PIM scale were moderate (κ = 0.459 and 0732). The consistency between the 2023 STOPP criteria and the 2024 Chinese criteria was moderate (κ = 0.448), but with the GO-PIM scale was poor (κ = 0.345). The results of multiple logistic regression analysis showed that polypharmacy was the main influencing factor for the occurrence of PIMs in all criteria (*P*<.001).

## 1. Introduction

In recent years, the incidence and mortality of malignant tumors in China have continued to rise, making cancer a significant public health problem. Annual medical expenses due to malignant tumors exceed 220 billion yuan, significantly increasing the societal economic burden. In 2022, there were 4.8247 million new cancer cases in China, concentrated in the 80 to 84 age group, with the average annual increase in the standardized incidence rate for all cancers being 1.4%.^[1]^ Cancer patients often undergo multiple treatment modalities, including not only traditional chemotherapy drugs but also targeted therapy drugs, immunotherapy drugs, and other types, leading to complex medication regimens. Additionally, older adults often suffer from multiple chronic diseases, further increasing the number of concomitant medications, thereby raising the risk of adverse drug events (ADEs) in older people living with cancer.

potentially inappropriate medications (PIMs) stand for PIMs and refer to situations where the risk of ADEs outweigh the potential benefits, and there is a lack of safer alternative medications.^[2-6]^ PIMs were significantly associated with all-cause mortality in older adult patients living with cancer and found that the mortality rate increases by 43% in older adult patients living with cancer with PIMs use.^[7]^ To guide rational medication use in older adults, many explicit PIM screening tools have been developed internationally. However, due to differences in medical practices, drug availability, and pharmacogenetics in specific populations, these criteria need to be adjusted according to the specific circumstances of each country.^[8]^ The Beers criteria was first proposed by American geriatric expert Beers in 1991, which has undergone multiple updates and plays a key role in preventing adverse drug reactions, evaluating drug use, and reducing the economic burden on patients. Currently, the American Geriatrics Society (AGS) released the latest Beers criteria in 2023, which proposes a clearer and more comprehensive rational medication plan for the elderly.^[9]^ The 2023 Screening Tool of Older Persons’ Prescription (STOPP) criteria, based on the latest research results and clinical evidence on rational medication use in older adults, added, revised, and deleted some criteria on the basis of the second edition, further expanding to 133 STOPP items.^[10]^ The 2024 Chinese criteria drew on the development method of the Beers criteria, comprehensively referenced 6 international PIM judgment criteria. Compared to the 2017 Chinese criteria, significantly added content includes recommendations for dose reduction or avoidance based on renal function, drug interactions, and alternative therapies.^[11]^ In addition, the NCCN Clinical Practice Guidelines in Oncology for Older Adult Oncology provided a list of commonly used supportive care drugs for older adult patients living with cancer. Hshieh et al translated this list into the geriatric oncology potentially inappropriate medication (GO-PIM) scale to quantify PIMs in older adult patients living with cancer with blood cancers.^[12]^ This was the first time the medication list in the NCCN guidelines has been used to screen for PIMs in cancer patients. Compared with other screening tools, the GO-PIM scale is updated regularly by oncologists and geriatricians. It not only formulates corresponding medication plans based on the actual situation of cancer patients but also provides alternative treatment suggestions to help patients with personalized medication management.

To date, there have been few studies evaluating PIMs in older adult patients living with cancer based on 4 criteria. Therefore, the aims of the present study were: to detect PIMs use by the 2023 AGS/Beers criteria, the 2023 STOPP criteria, the GO-PIM scale, and the 2024 Chinese criteria in older adult patients living with cancer; to compare the prevalence of PIMs and the accordance between the 4 PIM criteria; and to explore related risk factors for PIMs according to the 4 criteria.

## 2. Materials and methods

### 2.1. Study population and ethical considerations

This was a retrospective, single-center, cross-sectional study, and was reported in accordance with the Strengthening Reporting of Observational Studies in Epidemiology statement. Medical record data of older adult patients living with cancer hospitalized in the Department of Oncology at Yingshang County People’s Hospital from January to December 2023 were retrospectively collected and analyzed. Inclusion criteria: age ≥65 years; diagnosed with at least one malignant tumor, such as lung cancer, ovarian cancer, breast cancer, etc. Patients’ current diagnosis were classified using the International Classification of Diseases-11 (ICD-11). Exclusion criteria: length of hospital stay ≤2 days; Patients who died during hospitalization; and patients who did not use drugs or had incomplete data records. If the study subject was hospitalized multiple times, only the medical record data from the first hospitalization were included. The study protocol was approved by the Ethics Committee of Yingshang People’s Hospital (No. 2022011).

### 2.2. Data collection and evaluation of PIMs

The statistical data collected in this study included age, gender, clinical disease diagnosis, length of hospital stay, medication use, biochemical test results, etc. PIMs use was evaluated according to 4 different criteria, including the 2023 AGS/Beers criteria, 2023 STOPP criteria, GO-PIM scale, and 2024 Chinese criteria. The 2023 AGS/Beers criteria include: PIMs related to drugs; PIMs related to diseases or symptoms; PIMs for drugs that should be used cautiously in older adults; PIMs for drug interactions that should be avoided in older adults; and PIMs for dose reduction or avoidance based on renal function. The 2023 STOPP criteria are classified according to indications for use and drug effects, with a total of 13 categories and 133 PIMs recommendations. The GO-PIM scale includes drugs from different categories such as corticosteroids, benzodiazepines, first-generation antihistamines, etc. The 2024 Chinese criteria include a total of 154 items (a 57% increase compared to the 2017 version), including: “Criteria for Determining Potentially Inappropriate Medication Use in Chinese Older Adults under Non-Specific Conditions,” which includes 13 categories and 100 kinds/classes of drugs; “Criteria for Determining Potentially Inappropriate Medication Use in Chinese Older Adults under Specific Conditions,” which includes 56 kinds/classes of drugs applied under 54 specific conditions. Two authors were responsible for analyzing the medication use of the included patients, with double-checking. Disagreements were resolved through discussion to reach a consensus. Meeting any one of the 4 criteria indicated that the patient had PIMs. When the same patient had multiple PIMs, they were counted cumulatively.

### 2.3. Statistical analysis

Statistical analyses were performed using software packages IBM SPSS Statistics version 22.0 (IBM Corp., Armonk). When data followed a normal distribution, measurement data were expressed as mean ± SD, and comparisons between groups were performed using independent samples *t*-test. When data did not follow a normal distribution, they were expressed as median (interquartile range), and data were analyzed using the Mann–Whitney *U* test. Count data were expressed as frequencies and percentages, and comparisons between the group of 4 criteria were carried out by means of the Pearson χ^2^ test. Pairwise comparisons of multiple sample rates were analyzed using the Bonferroni method. The Kappa test was used to analyze the consistency of PIM screening results among the 4 criteria (κ value ≥ 0.75 indicated good consistency, 0.40 ≤ κ < 0.75 indicated moderate consistency; κ < 0.04 indicated poor consistency). Multivariate logistic regression analysis was used to screen for influencing factors of PIMs. Statistical significance was defined as *P* < .05 for all tests.

## 3. Results

### 3.1. Characteristics of the study population

A total of 484 older adult patients living with cancer were included in this study and 58.06% of patients were males. The median age of the patients and the average length of hospital stay were 72 (IQR: 68.25, 76) and 6 (IQR: 4, 11). The median number of disease diagnoses and medications were 5 (4, 7) and 7 (4, 12). 25.62% of the patients had lung cancer and the main treatment modality was symptomatic supportive treatment (64.05%). The main comorbid chronic disease was hypertension (26.86%; Table 1).

**Table 1:** Baseline characteristics of the patients.

	2023 AGS/Beers criteria			2023 STOPP criteria			GO-PIM scale			2024 Chinese criteria		
Characteristics	PIM	Non-PIM	*P* value*	PIM	Non-PIM	*P* value*	PIM	Non-PIM	*P* value*	PIM	Non-PIM	*P* value*
N (%)	302 (62.40)	182 (37.60)	<.001	204 (42.15)	280 (57.85)		316 (65.29)	168 (34.71)		344 (71.07)	140 (28.93)	
Sex			.217			.403			.101			.283
Male	182	99		123	158		192	89		205	76	
Female	120	83		81	122		124	79		139	64	
Age [M(P25, P75)]	72 (69.00, 76.25)	71 (68.00, 76.00)	.103	72 (68.00, 77.00)	71 (68.00, 76.00)	.276	72 (69.00, 76.00)	71 (68.00, 76.00)	.110	72 (69.00, 77.00)	70.5 (67.00, 75.75)	.055
Length of hospital stay [M(P25, P75)]	8 (5, 13)	4 (3, 6)	<.001	9 (5, 14)	5 (3, 7)	<.001	7 (5, 13)	4 (3.6)	<.001	7 (5, 13)	4 (3, 5)	<.001
Number of diagnoses [M(P25, P75)]	6 (4, 7)	4 (3, 5)	<.001	6 (4.25, 8)	4 (3, 6)	<.001	6 (4, 7)	4 (3, 5)	<.001	6 (4, 7)	4 (3, 5)	<.001
Number of medications [M(P235, P75)]	10 (7, 14)	3 (2, 6)	<.001	11 (7, 15)	5 (3, 9)	<.001	10 (7, 14)	3 (2, 5)	<.001	10 (7, 13)	3 (2, 5)	<.001
Metastasis	167	62	<.001	117	112	<.001	168	61	<.001	182	93	.006
Treatment method			.008			.04			<.001			.002
Antitumor drug therapy	115	48		56	107		126	37		130	33	
Radiotherapy	9	2		6	5		10	1		10	1	
Symptomatic and supportive treatment	178	132		142	168		180	130		204	106	
Cancer type			.022			.040			.993			.042
Lung cancer	88	36		62	62		81	43		97	27	
Esophageal cancer	44	39		33	50		49	34		52	31	
Colorectal cancer	34	24	.527	12	46	<.001	37	21	.799	38	20	.32
Comorbidity												
Hypertension	90	40	.060	75	55	<.001	94	36	.049	107	23	.001
Cerebrovascular disease	39	17	.234	38	18	<.001	43	13	.055	46	10	.052
Type 2 diabetes	37	9	.008	26	20	.038	35	11	.106	40	6	.013

GO-PIM = geriatric oncology potentially inappropriate medications, PIM = potentially inappropriate medication, STOPP = Screening Tool of Older Persons’ Prescriptions.

* Pearson χ^2^ or Mann–Whitney *U* tests were used to evaluate the differences between 4 criteria’s.

### 3.2. Quantity distribution of PIMs according to 4 criteria

As shown in Table 1, the prevalence of at least one PIM was 62.40%, 42.15%, 65.29%, and 71.07%, according to the 2023 AGS/Beers criteria, the 2023 STOPP criteria, the GO-PIM scale, and the 2024 Chinese criteria, respectively. Among patients with PIMs, the majority received 1 PIM, followed by 2 PIMs, and finally more than 3 PIMs (Table 2).

**Table 2:** Quantity distribution of PIMs according to 4 criteria.

	Total PIMs	1 PIM, n (%)	2 PIM, n (%)	≥3 PIM, n (%)	*P* value*
2023 AGS/Beers criteria	302	140 (46.36)	72 (23.84)	90 (29.80)	<.001
2023 STOPP criteria	204	118 (57.84)	51 (25.00)^a,b^	35 (17.16)^a^	
GO-PIM scale	316	98 (31.01)^a,^^b^	104 (32.91)	114 (36.08)^b^	
2024 Chinese criteria	344	61 (17.73)^a,b,c^	119 (34.59)^a^	164 (47.67)^a,b,c^	

GO-PIM = geriatric oncology potentially inappropriate medications, PIM = potentially inappropriate medication, STOPP = Screening Tool of Older Persons’ Prescriptions.

* Bonferroni method was used to evaluate the pairwise comparisons of 4 criteria’s.

a P < 0.05 compared with the 2023 AGS Beers criteria.

b P < 0.05 compared with the 2023 STOPP criteria.

c P < 0.05 compared with the GO-PIM scale.

### 3.3. Main medications in 4 sets of PIMs criteria

Table 3 shows main medications in 4 sets of PIMs criteria. According to the 2023 AGS/Beers criteria, 2023 STOPP criteria, the GO-PIM scale, and 2024 Chinese criteria, the most frequently used medications were metoclopramide (24.28%), megestrol acetate (23.48%), cimetidine (34.32%), and cimetidine (22.93%), respectively. Despite differences among PIM-related drugs screened by the 4 criteria, metoclopramide, megestrol acetate, cimetidine, and proton pump inhibitors (PPIs) all ranked in the top 5 at least 2 criteria.

**Table 3:** Top 5 PIMs according to 4 criteria.

	1		2		3		4		5	
	Drugs	N (%)	Drugs	N (%)	Drugs	N (%)	Drugs	N (%)	Drugs	N (%)
2023 AGS/Beers criteria (n = 663)	Metoclopramide	161 (24.28)	Diuretics	124 (18.70)	Medroxyprogesterone	81 (12.22)	PPI	60 (9.05)	Cimetidine and theophylline	37 (5.58)
2023 STOPP criteria (n = 345)	Medroxyprogesterone	81 (23.48)	PPI	60 (17.39)	NSAIDs are used for patients with severe hypertension	50 (14.49)	Using 2 NSAIDs simultaneously	48 (13.91)	Simultaneous use of 2 opioid analgesics	24 (6.96)
GO-PIM scale (n = 711)	Cimetidine	244 (34.32)	Metoclopramide	161 (22.64)	Dexamethasone	151 (21.24)	Oxycodone	45 (6.33)	Tramadol	33 (4.64)
2024 Chinese criteria (n = 1064)	Cimetidine	244 (22.93)	Metoclopramide	161 (15.13)	Theophylline	91 (8.55)	Medroxyprogesterone	81 (7.61)	PPI	60 (5.64)

GO-PIM = geriatric oncology potentially inappropriate medications, NSAID = non-steroidal anti-inflammatory drug; PIM = potentially inappropriate medication, PPI = proton pump inhibitor, STOPP = Screening Tool of Older Persons’ Prescriptions.

### 3.4. Comparison of concordance of PIMs evaluated between the 4 criteria

Table 4 shows concordance between the 4 criteria. The kappa statistics for the 2024 Chinese criteria with the 2023 AGS/Beers criteria and the GO-PIM scale indicated a good concordance (κ = 0.788 and 0810). The consistency between the 2023 AGS/Beers criteria and the 2023 STOPP criteria, the 2023 AGS/Beers criteria and the GO-PIM scale, the 2023 STOPP criteria and the 2024 Chinese criteria were moderate (κ = 0.459, 0732, and 0.448). However, the concordance between the 2023 STOPP criteria and the GO-PIM scale was poor (κ = 0.345).

**Table 4:** Comparison of concordance of PIMs evaluated.

	2023 AGS/Beers criteria		2023 STOPP criteria		GO-PIM scale		2024 Chinese criteria	
	κ	*P* value	κ	*P* value	κ	*P* value	κ	*P* value
2023 AGS/Beers criteria	–	–	0.459	<.001	0.732	<.001	0.788	<.001
2023 STOPP criteria	0.459	<.001	–	–	0.345	<.001	0.448	<.001
GO-PIM scale	0.732	<.001	0.345	<.001	–	–	0.810	<.001
2024 Chinese criteria	0.788	<.001	0.448	<.001	0.810	<.001	–	–

κ, kappa coefficient; P, probability value, based on κ test.

GO-PIM = geriatric oncology potentially inappropriate medications, STOPP = Screening Tool of Older Persons’ Prescriptions.

### 3.5. Factors associated with PIMs use

Univariate analysis showed significant differences in the incidence of PIMs according to the 4 criteria (*P* < .001). According to the 2023 AGS/Beers criteria, length of hospital stay, number of disease diagnoses, number of medications, presence of metastasis, treatment modality, lung cancer, and type 2 diabetes were statistically significant for PIMs occurrence. According to the 2023 STOPP criteria, length of hospital stay, number of disease diagnoses, number of medications, presence of metastasis, treatment modality, lung cancer, colorectal cancer, hypertension, cerebrovascular disease, and type 2 diabetes were statistically significant. According to the GO-PIM scale, length of hospital stay, number of disease diagnoses, number of medications, presence of metastasis, treatment modality, and hypertension were statistically significant. According to the 2024 Chinese criteria, length of hospital stay, number of disease diagnoses, number of medications, presence of metastasis, treatment modality, lung cancer, hypertension, and type 2 diabetes were statistically significant.

As shown in Table 5, Multivariate logistic regression analysis showed that according to the 2023 AGS/Beers criteria, age and number of medications were statistically significant for PIMs occurrence. According to the 2023 STOPP criteria, number of medications, treatment modality, colorectal cancer, and hypertension were statistically significant. According to the GO-PIM scale, age and number of medications were statistically significant. According to the 2024 Chinese criteria, age, number of medications, and hypertension were statistically significant. Among these, the number of medications was statistically significant for PIMs occurrence across all 4 criteria.

**Table 5:** The association between various factors and PIMs use.

Characteristics	2023 AGS/Beers criteria		2023 STOPP criteria		GO-PIM scale		2024 Chinese criteria	
	OR (95% CI)	*P* value*	OR (95% CI)	*P* value*	OR (95% CI)	*P* value*	OR (95% CI)	*P* value*
Sex	0.762 (0.440–1.318)	.331	0.831 (0.507–1.363)	.464	0.987 (0.572–1.705)	.964	1.442 (0.779–2.668)	.244
Age	1.053 (1.002–1.106)	.041	1.007 (0.964–1.052)	.759	1.06 (1.009–1.114)	.020	1.101 (1.039–1.167)	.001
Length of hospital stay	1.003 (0.951–1.059)	.904	1.008 (0.967–1.050)	.718	0.991 (0.938–1.047)	.743	1.061 (0.982–1.147)	.132
Number of diagnoses	0.913 (0.772–1.079)	.287	0.939 (0.821–1.074)	.361	0.968 (0.816–1.148)	.709	0.83 (0.683–1.008)	.061
Prescribed medications	1.682 (1.505–1.879)	<.001	1.356 (1.259–1.461)	<.001	1.649 (1.475–1.843)	<.001	1.907 (1.646–2.21)	<.001
Treatment method	1.093 (0.816–1.463)	.550	1.923 (1.458–2.535)	<.001	0.809 (0.605–1.081)	.152	1.443 (0.734–2.837)	.287
Metastasis	1.527 (0.839–2.777)	.166	1.57 (0.942–2.615)	.083	1.186 (0.650–2.164)	.578	1.022 (0.734–1.423)	.897
Lung cancer	1.944 (0.986–3.834)	.055	1.301 (0.730–2.321)	.372	0.739 (0.376–1.453)	.381	1.679 (0.786–3.586)	.181
Esophageal cancer	0.626 (0.286–1.374)	.243	0.892 (0.444–1.793)	.748	0.571 (0.262–1.244)	.158	0.551 (0.23–1.32)	.181
Colorectal cancer	0.936 (0.412–2.126)	.874	0.391 (0.170–0.900)	.027	0.692 (0.298–1.606)	.392	0.668 (0.266–1.675)	.389
Hypertension	1.57 (0.827–2.979)	.168	3.131 (1.794–5.464)	<.001	1.593 (0.829–3.059)	.162	3.297 (1.553–6.999)	.002
Cerebrovascular disease	0.574 (0.239–1.377)	.214	2.174 (1.015–4.658)	.056	1.027 (0.418–2.518)	.954	0.887 (0.32–2.459)	.818
Type 2 diabetes	1.719 (0.636–4.646)	.286	(0.379–1.881)	.679	0.772 (0.292–2.041)	.602	1.199 (0.361–3.982)	.767

CI = confidence interval, GO-PIM = geriatric oncology potentially inappropriate medications, OR = odds ratio, PIM = potentially inappropriate medication, STOPP = Screening Tool of Older Persons’ Prescriptions.

* Multivariate logistic regression was used to evaluate the risk factors of 4 criteria’s.

## 4. Discussion

Since cancer patients require radiotherapy, chemotherapy, molecular targeted therapy, and immunotherapy, and considering the significant toxicity of anticancer drugs, some drugs are used simultaneously to prevent adverse reactions caused by anticancer drugs, thus making medication use more complex and significantly increasing the risk of PIMs occurrence.^[13-16]^ In the present study, we found a high incidence of PIMs in older adult patients living with cancer. The incidence of PIMs screened by the 2024 Chinese criteria was the highest (71.07%), while that screened by the 2023 STOPP criteria was the lowest (42.15%), suggesting that the 2024 Chinese criteria may be more sensitive for screening PIMs in Chinese older adult patients living with cancer. A meta-analysis reported that the prevalence of PIMs use in older adult patients living with cancer ranged from 10.8% to 57.5%.^[7]^ Compared with a published study on the prevalence of PIMs in older adult outpatients living with cancer (32.65%), our study found a higher prevalence of PIMs.^[17]^ This is mainly because inpatients often have more complex comorbidities than outpatients and require more medications for treatment. Furthermore, this study is the first to use the GO-PIM scale to identify PIMs in older adult patients living with cancer, which helps determine the suitability of drugs for cancer patients. Compared with other screening tools, the GO-PIM scale focuses more on the 2 specific areas of geriatrics and oncology. It not only lists drugs that should be used cautiously in older adult patients living with cancer but also provides specific medication suggestions and alternative treatment plans, thereby supporting personalized medication management for patients.

Our results show that according to the 2023 AGS/Beers criteria, 2023 STOPP criteria, GO-PIM scale, and 2024 Chinese criteria, the most common PIMs were metoclopramide (24.28%), megestrol acetate (23.48%), cimetidine (34.32%), and cimetidine (22.93%), respectively. Although the specific PIMs identified varied across the 4 criteria, metoclopramide, megestrol acetate, cimetidine, and PPIs all ranked in the top 5 according to at least 2 criteria. The higher incidence of PIMs screened by the 2024 Chinese criteria is mainly related to the use of cimetidine. Cimetidine was listed as a drug requiring intervention in the 2024 Chinese criteria, while in the 2023 AGS/Beers criteria, cimetidine was only considered PIMs when combined with theophylline. Since the participants of this study were older adult patients living with cancer, the use of anticancer drugs during treatment can cause adverse digestive system reactions such as nausea and vomiting, affecting the effectiveness of drug therapy. Cimetidine is an H_2_ receptor antagonist, mainly used to inhibit gastric acid secretion. It is commonly used to prevent anticancer drug-related gastrointestinal discomfort. However, its widespread use has led to growing concerns regarding off-label application. Additionally, cimetidine reduces gastric acid secretion by inhibiting the cytochrome P450 enzyme system in the liver, especially CYP3A4, and has more interactions than other H_2_ receptor antagonists. Therefore, in clinical practice, it is necessary to strictly master the clinical application indications of cimetidine to reduce medication risks. Besides cimetidine, drugs used to prevent gastrointestinal discomfort caused by anticancer drugs also include metoclopramide and PPIs. Metoclopramide has a strong central antiemetic effect but can cause extrapyramidal reactions, especially in older adults, fatigued, and debilitated patients. The GO-PIM scale believes that metoclopramide should be avoided, suggesting short-term use of corticosteroids or other antiemetics for patients. PPIs have a rapid onset, strong and long-lasting acid suppression effects, and are widely used clinically to preventively alleviate gastrointestinal discomfort caused by anticancer drugs. However, long-term use of PPIs may increase the risk of fractures, vitamin B_12_ deficiency, hypomagnesemia, and Clostridium difficile infection in older adults.^[18]^ Jenghua et al reported that 44.5% of inpatients received prophylactic antibiotics without a clear indication, a practice that not only consumes unnecessary healthcare resources but may also adversely affect patient outcomes.^[19]^ Megestrol acetate is a steroid hormone drug that shows significant efficacy in treating severe anorexia and cachexia symptoms often faced by cancer patients but increases the risk of thrombotic events and death in older adults.

The kappa statistics for the 2024 Chinese criteria with the 2023 AGS/Beers criteria and the GO-PIM scale indicated a good concordance. The consistency between the 2023 AGS/Beers criteria and the 2023 STOPP criteria, the 2023 AGS/Beers criteria and the GO-PIM scale, the 2023 STOPP criteria and the 2024 Chinese criteria were moderate. In addition, the 2023 STOPP criteria showed poor concordance with the GO-PIM scale. This is similar to another study conducted among older adult patients living with cancer in China.^[20]^ The reason may be differences in the drugs included in different criteria. For example, drugs screened by the Chinese criteria such as megestrol acetate, tramadol, and ibuprofen overlap significantly with the Beers criteria, and drugs like clopidogrel, warfarin, and nicergoline were added. The 4 criteria used in this study have certain advantages and limitations and can complement each other to some extent. AGS/Beers criteria was the first screening tool used to identify PIMs in older adults. The version has been updated multiple times and has been used as a reference by other criteria. Compared to the 2023 AGS/Beers criteria, the 2023 STOPP criteria focused more on PIMs under specific disease states, thus screening for fewer PIMs. The GO-PIM scale provides new research ideas for reviewing the suitability of medication use in older adult patients living with cancer, focusing mainly on commonly used supportive care medications, with relevant drugs and suggestions aligning with actual cancer treatment. The 2024 Chinese criteria was an updated version developed after a comprehensive review of authoritative international PIM criteria and the latest evidence from 2018 to 2023. The integration of 6 well-established PIM frameworks enhances the generalizability of the Chinese criteria for international comparisons while maintaining their relevance to the unique clinical context of China.^[21]^ The 2024 Chinese criteria only included studies that explicitly reported ADEs in people aged 65 or older, ensuring the applicability of the findings in older adults. The revisions involved adding new medications, removing outdated items, and updating recommendations, evidence levels, and risk points, resulting in a robust and clinically relevant PIMs list, significantly improving practicality and clinical guidance.

Multivariate logistic regression analysis showed that the number of medications was the main influencing factor for PIMs occurrence across all 4 criteria, consistent with the results reported in the literature.^[22-26]^ Since older adult patients living with cancer commonly have multiple chronic comorbidities, with more severe disease symptoms, different types of drugs are needed to control the condition. Additionally, while anticancer drugs treat cancer, they also bring many adverse reactions, requiring the simultaneous use of some drugs for pretreatment, making PIM a major medication safety issue. Therefore, when clinicians prescribe medications, they should strictly follow relevant clinical guidelines, combine the patient’s diseases and current medications, carefully evaluate the risks and benefits of drugs, reduce unnecessary drug use, and help reduce the risk of PIMs occurrence. Furthermore, according to the 2023 AGS/Beers criteria, GO-PIM scale, and 2024 Chinese criteria, age is a main influencing factor for PIMs occurrence, consistent with related studies.^[27-30]^ The analysis suggests that the physiological function of advanced-age patients severely declines, leading to reduced drug efficacy, slow drug metabolism, resulting in drug accumulation in the body. Coupled with the more common coexistence of multiple diseases and polypharmacy, adverse drug reactions are more likely to occur. This suggests that clinical attention should be highly paid to advanced-age cancer patients with multiple medications to ensure medication safety.

Our study has several limitations. First, this is a single-center study conducted in a tertiary hospital in China, so its results may not necessarily be applicable to other countries. Second, we collected patients’ medication information at admission, which may not accurately reflect the real medication situation of older adult patients living with cancer, as patients may have used other drugs before admission. Third, the association between the occurrence of PIMs and adverse clinical outcomes needs further study. Fourth, we only used 4 criteria to evaluate PIMs and did not use some other specific criteria, such as the OncPal criteria.^[31]^ However, these criteria may be more applicable to palliative care patients. Applying specific criteria to evaluate PIMs use in specific patients requires further research.

## 5. Recommendations for future research

Prospective prescription review plays a positive role in improving medical quality. It is recommended to optimize the judgment criteria for PIMs in older adults through informatization means to reduce medication risks. Studies have shown that after optimizing prospective prescription review, the incidence of PIMs in older adult patients significantly decreased, and it can promptly alert physicians, helping them quickly identify medication risks.^[32]^ Additionally, a multidisciplinary integration mechanism should be established, with a team consisting of doctors, nurses, and pharmacists participating in medication review to reduce drug expenditure and the risk of drug-induced diseases. Similar practices already exist in Germany with significant effects.^[33]^ Simultaneously, it is necessary to strengthen training for healthcare personnel on PIMs screening and management, develop standardized processes and assessment mechanisms, and provide timely feedback on management effectiveness. For the older adults, health education should be carried out, covering the occurrence of PIMs, adverse outcomes, the benefits of standardized medication use, and methods to improve self-efficacy, promoting medication safety.

## 6. Conclusion

In summary, this study used 4 criteria simultaneously to analyze PIMs in older adult patients living with cancer and found that most patients had PIMs. There were certain differences in the consistency of PIMs evaluation results among the 4 criteria, with the 2024 Chinese criteria screening for the highest incidence of PIMs. According to the 4 criteria, the number of medications was the main influencing factor for PIMs occurrence, suggesting that a comprehensive assessment is needed before medication use in older adult patients living with cancer to prevent inappropriate polypharmacy, thereby reducing the occurrence of adverse events. In clinical work, multiple criteria can be used in combination to evaluate PIMs in older adult patients living with cancer to effectively ensure medication safety.

## Acknowledgments

The authors gratefully thank Dong Wu for the statistical guidance at Fuyang People’s Hospital.

## Author contributions

**Conceptualization:** Yulong Liu, Qiao Su.

**Data curation:** Yulong Liu, Qiao Su.

**Methodology:** Xiaowu Wang, Dong Wu.

**Software:** Dong Wu.

**Supervision:** Xiaowu Wang.

**Validation:** Xiaodong Li.

**Writing – original draft:** Yulong Liu, Qiao Su.

**Writing – review & editing:** Xiaowu Wang.
